# Phylogenetic diversity and spatiotemporal dynamics of bacterial and microeukaryotic plankton communities in Gwangyang Bay of the Korean Peninsula

**DOI:** 10.1038/s41598-022-06624-7

**Published:** 2022-02-22

**Authors:** Dukki Han, Hanseob Shin, Ji-Hoon Lee, Chang-Keun Kang, Dong-Gyun Kim, Hor-Gil Hur

**Affiliations:** 1 Department of Marine Molecular Bioscience, Gangneung-Wonju National University, 7, Jukheon-gil, Gangneung-si, Gangwon-do 25457 Republic of Korea; 2 School of Earth Sciences and Environmental Engineering, Gwangju Institute of Science and Technology, 123 Cheomdangwagi-ro, Buk-gu, Gwangju, 61005 Republic of Korea; 3 Department of Bioenvironmental Chemistry, Jeonbuk National University, Jeonju, Republic of Korea; 4 Biotechnology Research Division, National Institute of Fisheries Science, Busan, 46083 Republic of Korea

**Keywords:** Microbial biooceanography, Water microbiology, Microbial ecology

## Abstract

Nutrient dynamics function globally, flowing from rivers to the ocean (estuarine–coastal zone), and are vulnerable to climate change. Microbial habitats can be affected by marine nutrient dynamics and may provide a clue to predict microbial responses to environmental heterogeneity in estuarine–coastal zones. We surveyed surface seawater in Gwangyang Bay, a semi-enclosed estuary in Korea, from 2016 to 2018 using a metabarcoding approach with prokaryotic 16S and eukaryotic 18S rRNA genes. Bacterial and microeukaryotic communities in these waters showed distinct local communities in response to environmental heterogeneity and community transition at spatiotemporal scales in the estuarine–coastal zone. The relative abundance of prokaryotic and eukaryotic operational taxonomic units suggested a microbial trophic interaction in the Gwangyang Bay waters. We found that the community assembly process in prokaryotic communities was primarily influenced by biological interaction (immigration–emigration), whereas that in eukaryotic communities was more affected by environmental stress (habitat specificity) rather than by biotic factors. Our findings in the Gwangyang Bay waters may provide information on underlying (biotic or abiotic) factors of the assembly process in microbial communities in the estuarine–coastal zone.

## Introduction

The estuarine–coastal transitional zone has a strong gradient of the salinity and organic matters from rivers to the ocean, and the organic-rich river waters contribute to nutrient cycling in the marine environment^1^. The marine nutrient cycle in the coastal zone is generally linked to river discharge and the subsequent mixing with coastal waters. The river discharge regulates freshwater input and organic matter contents, and such regulating factors vary seasonally in temperate estuaries^2–4^. The recent observation argued that the estuaries under the climate change may be more concerned than the early prediction^5^. Various information on the response of the estuarine–coastal zone to the climate change is required for more accurate prediction. Useful predictive models in the coastal system need to consider the effect of biological contribution as well as the physical and biogeochemical factors^6^. For example, climate-related changes in the marine environment and their possible impacts on biodiversity in vulnerable ecosystem deserve more consideration. In particular, the microbial contribution to marine nutrient dynamics is an important issue^7,8^, and numerous oceanic surveys have been performed to increase the understanding of microbial habitats in marine environments^9–18^.

Gwangyang Bay (GB), a semi-enclosed estuary, forms the estuarine–coastal zone at the southern tip of the Korean Peninsula and is a suitable area to monitor the spatiotemporal variability of environmental heterogeneity in the estuarine–coastal zone^19–21^. Furthermore, a recent metabarcoding survey of GB revealed that water mass mixing shapes bacterial communities in the estuarine–coastal zone and provided valuable insights into bacterial contributions to phytoplankton-derived organic matter under seasonal variation and phylogenetic bacterial diversity at euphotic depths^16^. Although the previous GB survey by Han et al.^16^ improved our understanding of the phylogenetic structuring (phylogenetic over-dispersion or clustering) of bacterial communities in the estuarine–coastal zone, the ecological significance of eukaryotic communities and its comparison with that of prokaryotic communities remains unknown. However, considering the preliminary findings of Han et al.^16^, GB may provide fundamental information regarding the phylogenetic responses of microbial (prokaryotic and eukaryotic) communities to the seasonal climate change in the estuarine–coastal zone.

The phylogenetic turnover of local communities is estimated using the net relatedness index (NRI), nearest taxon index (NTI), and β-nearest taxon index (βNTI)^22,23^. Briefly, NRI and NTI can determine the influence of biotic and abiotic factors on community assembly with two phylogenetic structuring patterns: (1) phylogenetic over-dispersion (biotic interaction with immigration–emigration) and (2) phylogenetic clustering (abiotic interaction with habitat specificity). In addition, βNTI can be used to predict the relative influence of deterministic (environmental selection) and stochastic (ecological drift or dispersal ability) processes in microbial assemblages in various environments^23–29^.

The present study primarily aimed to estimate the phylogenetic diversity of prokaryotic and eukaryotic communities and their ecological significance at the spatiotemporal scale in the estuarine–coastal zone. The microbial diversity and community composition in surface seawater (< 0.3 m depth) in GB were surveyed using a metabarcoding approach with prokaryotic 16S and eukaryotic 18S rRNA genes. Furthermore, the phylogenetic diversity was estimated using ecological statistics with metabarcoding sequences.

## Results

### Environmental heterogeneity in the estuarine–coastal zone

**Figure 1:**
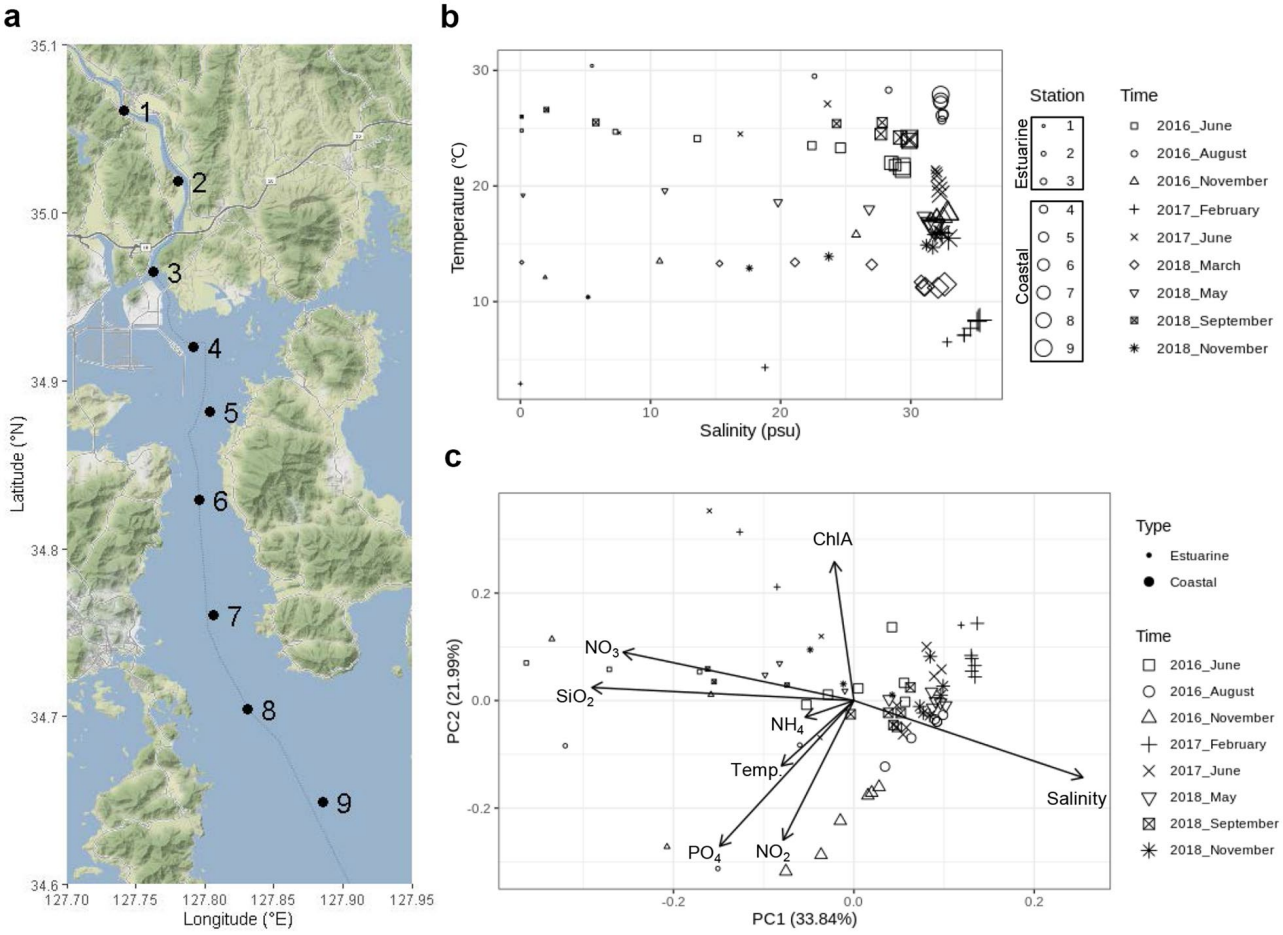
Environmental heterogeneity in GB waters. (**a**) Sampling stations were drawn by Google Map using the ggmap package^58^ in R. (**b**) Spatiotemporal variation of GB waters in T-S diagram and (**c**) its statistical separation with environmental factors supported by principal component analysis. Values of environmental parameters in this study were listed in Supplementary Informationn 1 (excel file).

Nine sampling stations in GB (stations 1–9) were selected. The endpoint of water mass mixing between stations 3 and 4 was determined on the basis of their geographic locations (Fig. 1a), and the temperature-salinity gradient (Fig. 1b) driven by the mixing of water masses was analyzed as previously reported^16^. GB metadata were assigned to two water types such as estuarine (stations 1–3) and coastal (stations 4–9) according to the previous separation of GB waters^16^, and their spatiotemporal distribution was explained using principal component analysis (PCA) with water mass properties (temperature, salinity, PO_4_, NH_4_, NO_2_, NO_3_, SiO_2_, and ChlA). In the present study, environmental heterogeneity under the extended temporal scale against the previous study^16^ revealed the similar PCA pattern (Fig. 1c) to the previous pattern^16^, indicating the consistence of seasonal water mass mixing in GW. Permutational multivariate analysis of variance (PERMANOVA) supported the spatiotemporal separations of PCA with a significance level of R^2^ = 0.46 and *P* < 0.01 between estuarine and coastal types and of R^2^ = 0.28 and *P* < 0.01 among sampling times (Table S1). The post-hoc PERMANOVA showed that most of the pairwise comparisons of sampling times within the coastal type were significantly different (*P* < 0.01), whereas the comparisons of sampling times within the estuarine type were not significant (*P* > 0.01) (Table S1).

### Microbial diversity and community composition at the spatiotemporal scale

**Figure 2:**
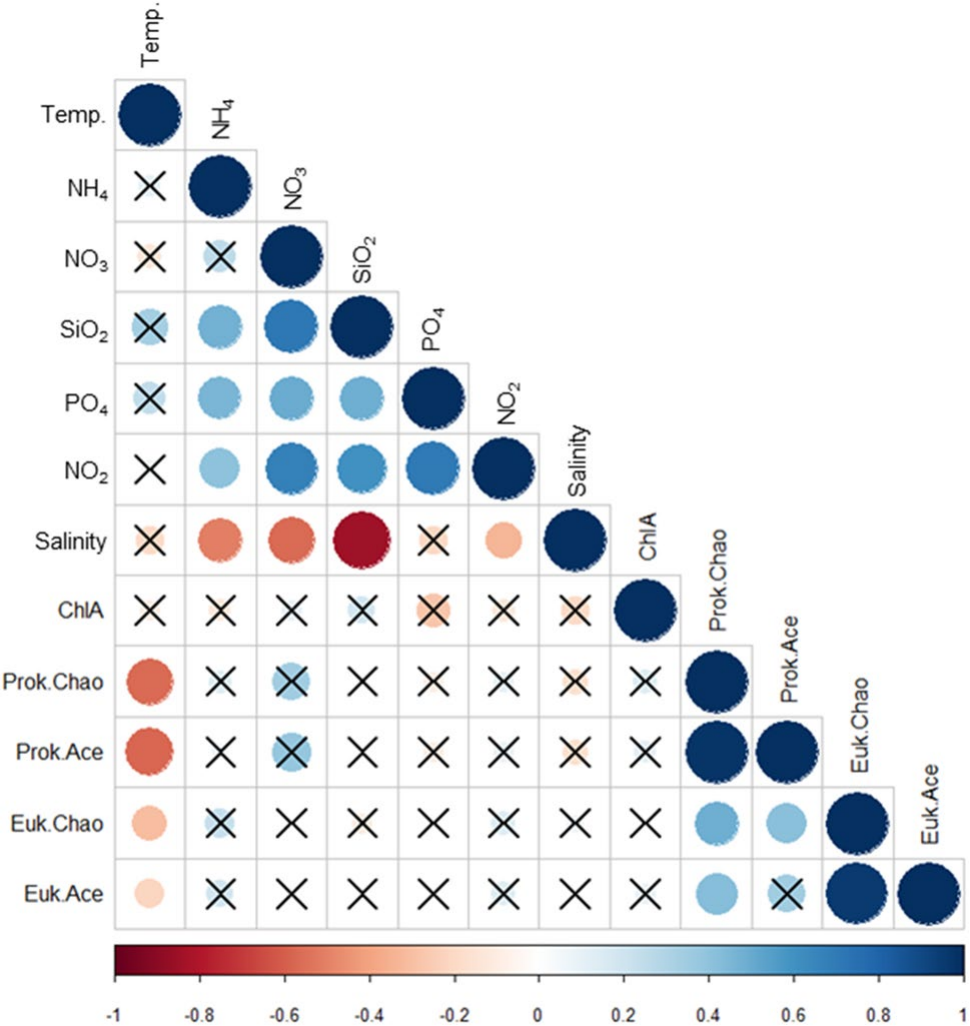
Correlation between indices of microbial alpha diversity and environmental parameters. Correlation coefficients are colored according to the value scale. Positive correlations are displayed in a blue scale while negative correlations are displayed in a red scale. The insignificant coefficients are marked according to the *P* value (*P* < 0.05).

A total of 1,808,000 sequences (1,027,000 sequences of prokaryotic 16S rRNA gene from 79 samples and 781,000 sequences of eukaryotic 18S rRNA gene from 71 samples) were obtained from GB waters. These sequences were independently clustered into 6092 prokaryotic and 2693 eukaryotic operational taxonomic units (OTUs) to analyze microbial diversity (alpha and beta) and community composition. The indices of alpha diversity (species richness) in both prokaryotes and eukaryotes showed relatively similar distributions in estuarine and coastal types and moved up and down the temporal scale periodically (Fig. S1). This alpha diversity distribution revealed a significant negative correlation with temperature change in the GB (*P* < 0.05). In particular, the prokaryotic indices of Chao and Ace were more strongly correlated (Chao: − 0.56 and Ace: − 0.57) than the eukaryotic indices (Chao: − 0.30 and Ace: − 0.22) (Fig. 2). Non-metric multidimensional scaling (NMDS) used to view microbial beta diversity (Fig. S2) revealed the community transitions of prokaryotes and eukaryotes in GB waters at the spatiotemporal scale. The beta diversity patterns in prokaryotes and eukaryotes were statistically supported by analysis of molecular variance (AMOVA) (Table S2). The spatial separation between estuarine and coastal types was significant in both prokaryotic (*P* < 0.01) and eukaryotic (*P* < 0.01) communities, and in the temporal separation, the two communities showed a significant difference among the sampled months (*P* < 0.01). Particularly, in prokaryotic communities, there were significant differences in all pairwise comparisons, except in the pair of March and November 2018 (*P* > 0.01). Similarly, eukaryotic communities revealed significant differences in most pairs of temporal comparisons, except in three pairs (*P* > 0.01; June 2017 and May 2018, March and May 2018, and May and November 2018). Overall, the beta diversity patterns indicated the existence of local prokaryotic and eukaryotic communities in response to the spatiotemporal separation in GB waters.

Microbial communities in GB waters are characterized by a few major prokaryotic and eukaryotic taxa (with a frequency of more than 1% in the sum of sequences) at the phylum level. Further, 99.96% of the prokaryotic sequences were bacterial sequences; however, there were only 0.04% archaeal sequences. Among the major prokaryotic taxa, Proteobacteria (48.71%) were predominant, followed by Bacteroidetes (19.65%), Actinobacteria (11.78%), Verrucomicrobiota (8.24%), Cyanobacteria (5.35%), Planctomycetota (2.22%), and Firmicutes (1.94%). Although the relative abundances of these prokaryotic taxa showed a variable distribution in GB waters, this variation indicated distinctive local communities, as shown by the beta diversity data (Fig. S3). Particularly, the distribution of heterotrophic Proteobacteria, Bacteroidetes, and Actinobacteria showed remarkable changes in response to the spatiotemporal separation. In contrast, eukaryotic sequences originated from phytoplankton (74.96%), zooplankton (18.66%), fungi (5.47%), fish (0.14%), and unknown eukaryotic sequences (5.47%). The eukaryotic communities comprised the following major eukaryotic taxa: Diatomea (35.69%, phytoplankton), Dinoflagellata (30.54%, phytoplankton or zooplankton), Ciliophora (16.01%, zooplankton), Ascomycota (3.63%, fungi), Cryptophyceae (3.55%, phytoplankton), Chlorophyta (2.70%, phytoplankton), Ochrophyta (2.26%, phytoplankton), and Cnidaria (1.42%, zooplankton). Similar to the relative abundance of prokaryotic taxa, that of these major eukaryotic taxa represented the distinctive local communities in GB waters, and the predominant phytoplankton populations (Diatomea and Dinoflagellata) revealed spatiotemporal variations (Fig. S3). Among these major prokaryotic and eukaryotic taxa, the relative abundance of Proteobacteria, Bacteroidetes, Actinobacteria, Firmicutes, Dinoflagellata, Chlorophyta, and Cnidaria was significantly correlated with the gradient of temperature or salinity (Table S3), and Actinobacteria showed a strong correlation with the salinity change (cor: − 0.63).

### Phylogenetic diversity patterns

**Figure 3:**
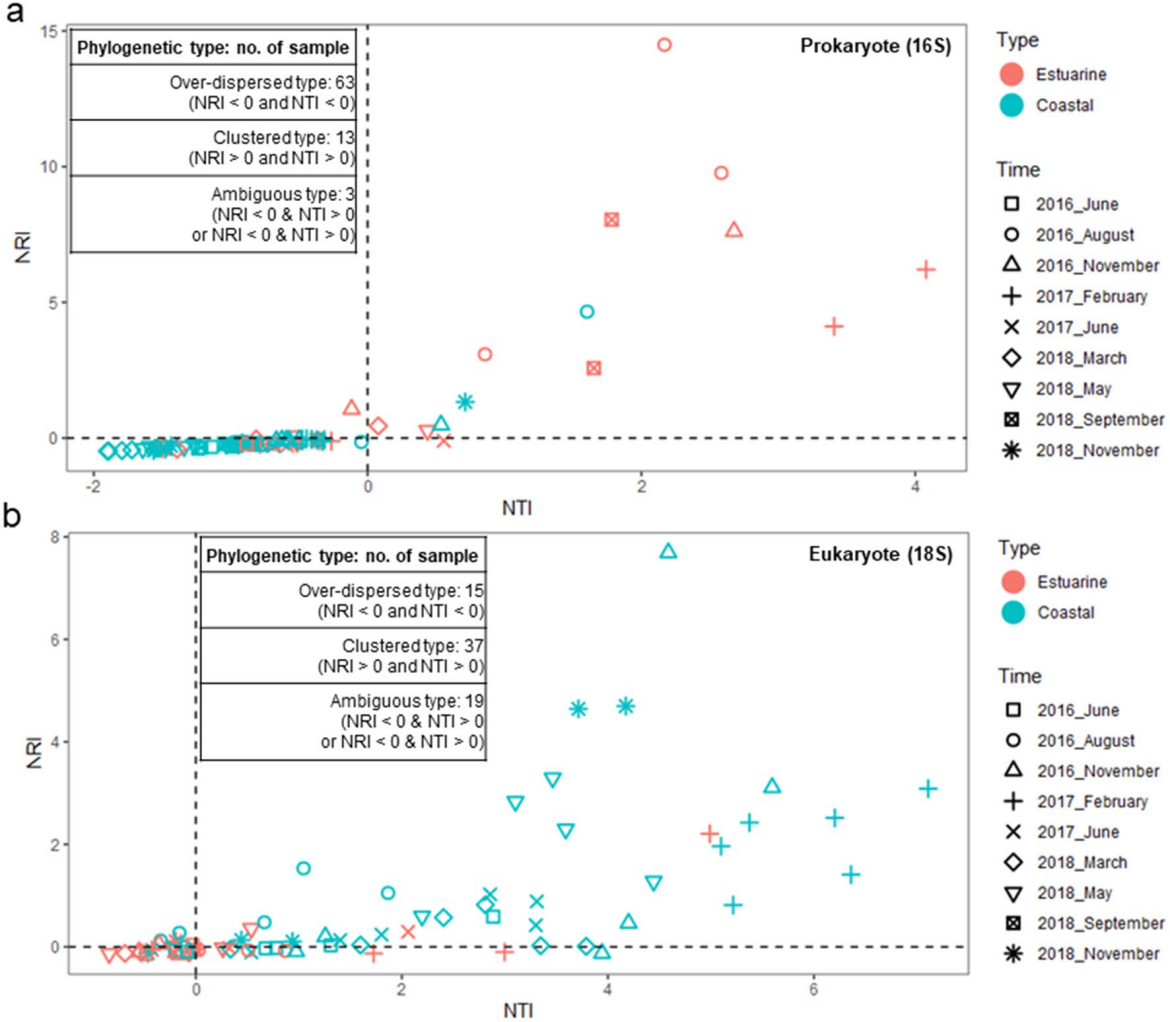
Phylogenetic turnover of microbial communities estimated using the Net Relatedness Index and Nearest Taxon Index. (**a**) Prokaryotic community and (**b**) eukaryotic community.

**Figure 4:**
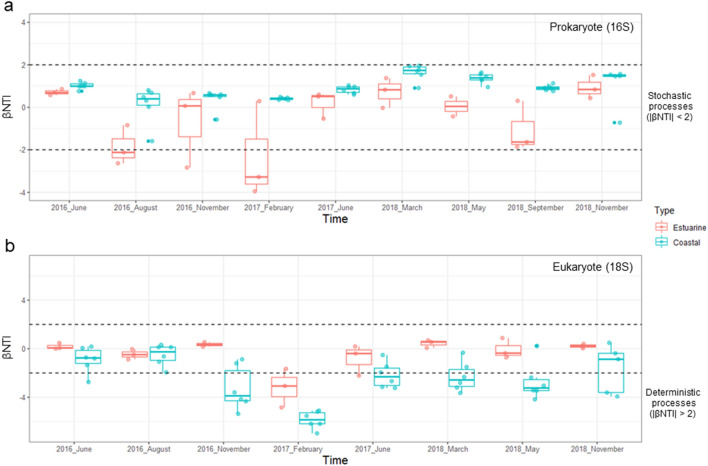
The relative influence of deterministic and stochastic processes in microbial communities using β-Nearest Taxon Index (βNTI). (**a**) Prokaryotic community and (**b**) eukaryotic community. βNTI values determine the relative influence of deterministic (|βNTI|> 2) and stochastic (|βNTI|< 2) processes on community assembly.

From the prokaryotic (n = 6092) and eukaryotic (n = 2693) OTUs, the 500 most abundant OTUs (accounting for 96.19% of total prokaryotic sequences and 97.29% of eukaryotic sequences) were selected for further calculation of NRI, NTI, and βNTI because of limited computing resources. Phylogenetic structuring patterns were determined using NRI and NTI values^16,30–32^: phylogenetic over-dispersion (NRI and NTI < 0), which implies the assembly of microbial communities under biological interactions, or phylogenetic clustering (NRI and NTI > 0) for the assembly process associated with abiotic factors. GB waters showed a distinguishable balance of phylogenetic structuring between prokaryotic and eukaryotic communities. For example, most prokaryotic communities from 79 GB waters (53 coastal and 26 estuarine samples) were classified as over-dispersed type (n = 63), followed by clustered (n = 13) and ambiguous type (n = 3; NRI < 0 and NTI > 0, NRI < 0 and NTI > 0) (Fig. 3a). Of prokaryotic communities, coastal waters (n = 53) were primarily classified as over-dispersed type (n = 50), whereas estuarine waters (n = 26) were in over-dispersed (n = 13) or clustered type (n = 10). In contrast, the eukaryotic patterns of phylogenetic structuring in 71 GB waters (47 coastal and 24 estuarine samples) comprised mostly of clustered type (n = 37), and the remaining waters were of the over-dispersed type (n = 15) or ambiguous type (n = 19) (Fig. 3b). Of eukaryotic communities, most of coastal waters (n = 47) were classified as clustered type (n = 34), whereas estuarine waters (n = 24) were similarly in over-dispersed (n = 11) and ambiguous type (n = 10). Interestingly, the predominant patterns of both prokaryotic (over-dispersed type) and eukaryotic (clustered type) communities occurred primarily in the GB coastal waters. βNTI values were calculated to determine the relative influence of deterministic (|βNTI|> 2) and stochastic (|βNTI|< 2) processes on community assembly^23,27^ in GB waters. The assembly of prokaryotic communities was primarily dominated by the stochastic process (Fig. 4a), whereas more than half of the coastal waters (53%) in eukaryotic communities were influenced by the deterministic process (Fig. 4b).

### Spatiotemporal distribution and microbial association network of prokaryotic and eukaryotic OTUs

**Figure 5:**
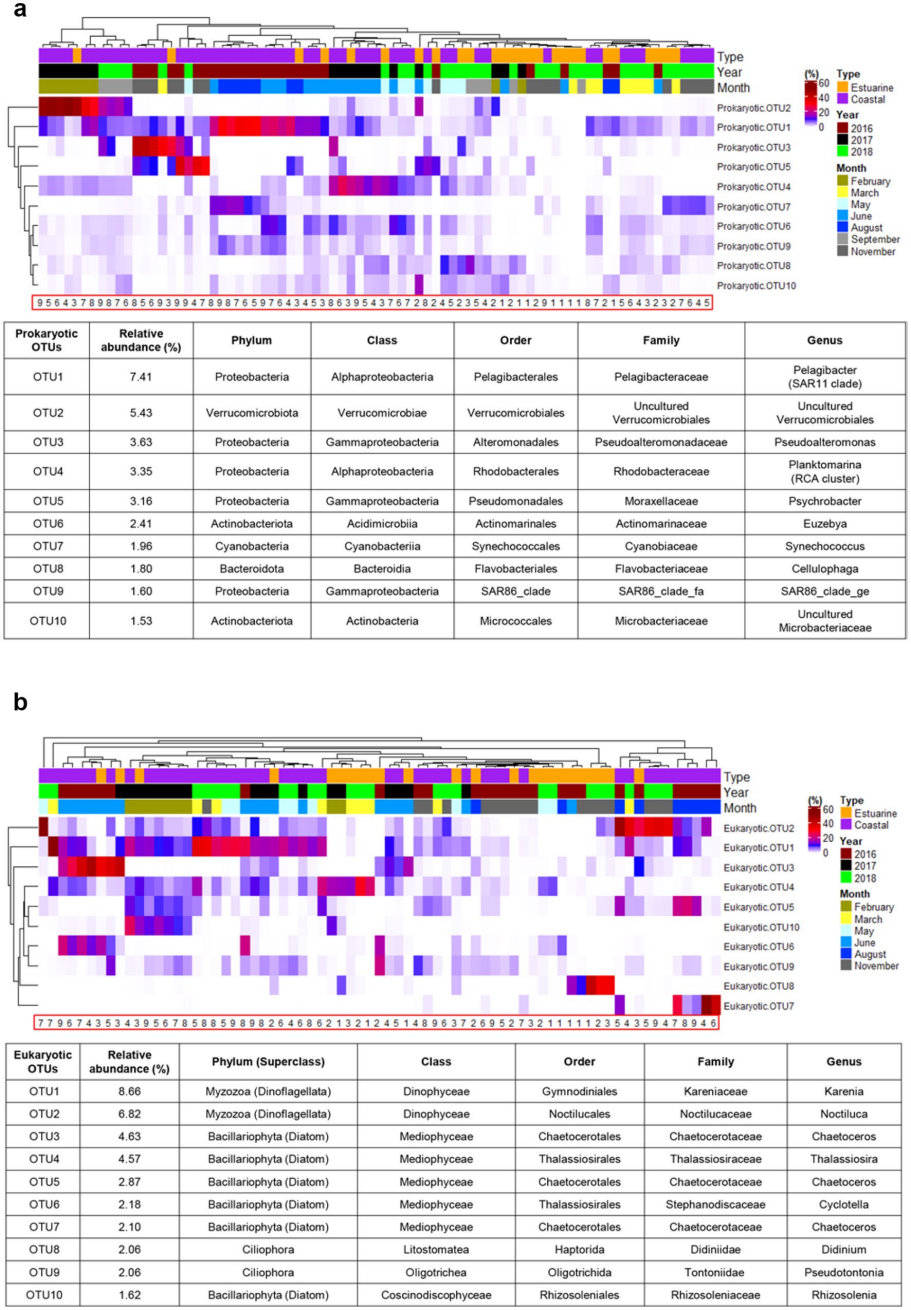
Heat-map visualization for distribution of microbial operational taxonomic units (OTUs). (**a**) Prokaryotic OTUs and (**b**) eukaryotic OTUs. Numbers in red line box indicate the GB stations.

The 10 most abundant prokaryotic and eukaryotic OTUs were selected from the total microbial OTUs, and their distribution was visualized using a heatmap with indicator species analysis. The taxonomy of prokaryotic and eukaryotic OTUs was identified against the NCBI database (https://www.ncbi.nlm.nih.gov/). Overall, the heatmap visualization of the 10 most abundant prokaryotic OTUs partially represented the spatiotemporal variation of GB waters (Fig. 5a). Particularly, prokaryotic OTU1, OTU6, OTU7, and OTU9 significantly occurred in coastal waters (indicator value > 0.6, *P* < 0.01); however, their distribution showed less or non-significant temporal (month) specificity (indicator value < 0.6, *P* > 0.01) (Table S4). The other prokaryotic OTUs had non-significant spatial (type) specificity (indicator value < 0.6, *P* > 0.01), whereas OTU2 and OTU5 significantly occurred in February 2017 and November 2016 (indicator value > 0.6, *P* < 0.01), respectively (Table S4). The 10 most abundant prokaryotic OTUs were taxonomically identified as Proteobacteria (OTU1, OTU3, OTU4, OTU5, and OTU9), Actinobacteria (OTU6 and OTU10), Verrucomicrobiota (OTU2), Cyanobacteria (OTU7), and Bacteroidetes (OTU8), and these taxonomies are all common bacteria in GB waters^16^.

**Figure 6:**
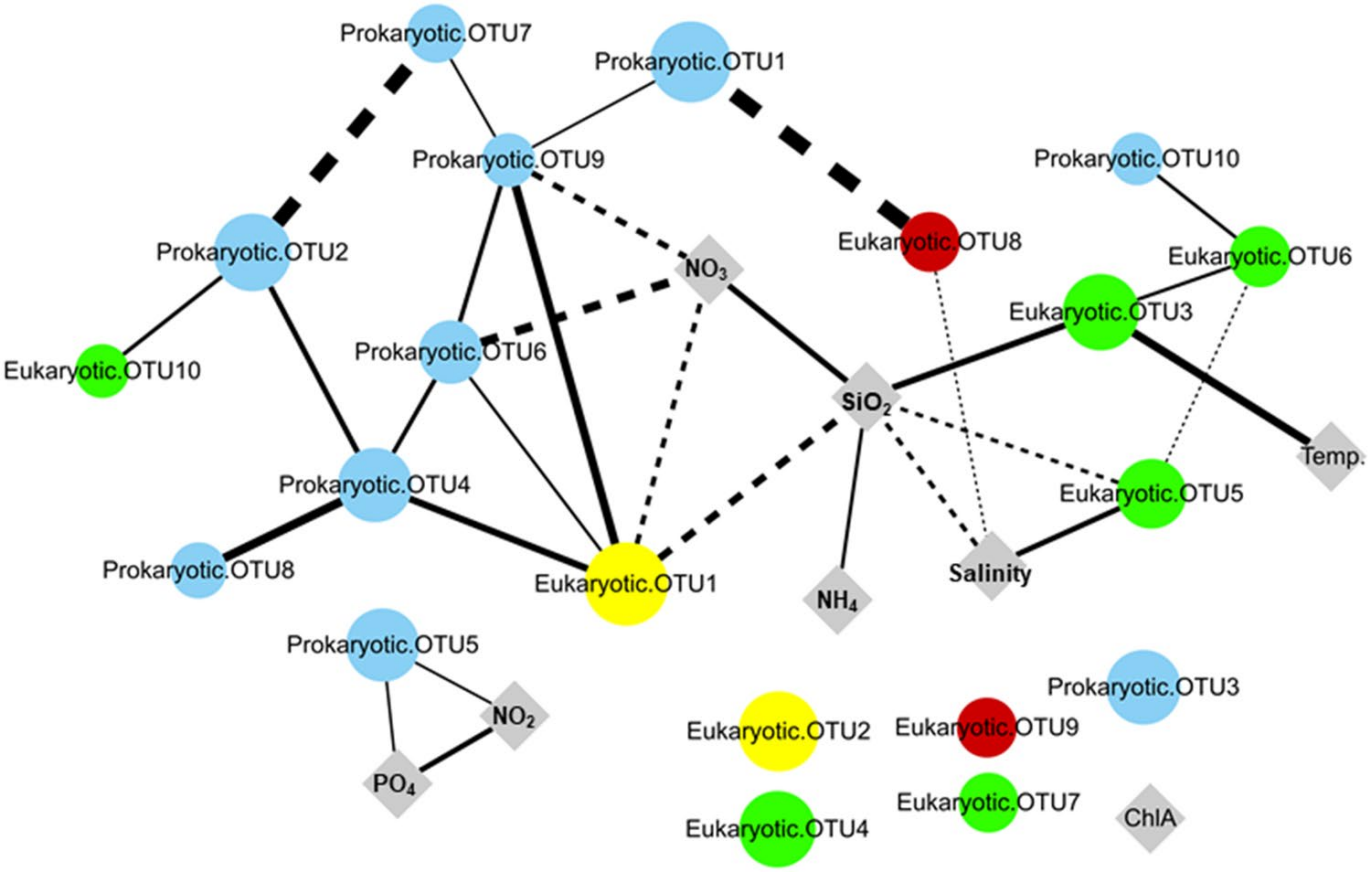
A microbial association network with environmental factors. In the network, each node represents the prokaryotic and eukaryotic OTUs in figure (blue circles: prokaryotic OTUs; green circles: Diatomea OTUs; yellow circles: Dinoflagellata OTUs; red circles: Ciliophora OTUs) or environmental factors in Fig. 1c (grey diamonds). The size of OTU nodes represents its sum of relative abundance, and the edges connecting the nodes (OTUs and environmental factors) represent their correlations. Of the edges, edge (line) thickness indicates the correlation coefficient (the thicker the line, the stronger correlation), and the solid lines and dotted lines represent positive and negative correlations, respectively. The isolated nodes with no edge indicate none of interaction at significant level (correlation coefficient < 0.5, *P* > 0.05).

Similar to the prokaryotic OTUs, the 10 most abundant eukaryotic OTUs showed the spatiotemporal variation in GB waters (Fig. 5b). For example, eukaryotic OTU1, OTU2, and OTU5 significantly occurred in coastal waters (indicator value > 0.6, *P* < 0.01) but was not specific for the temporal separation (indicator value < 0.6 or *P* > 0.01) (Table S4). In contrast, eukaryotic OTU8 was significantly distributed in estuarine waters (indicator value > 0.6, *P* < 0.01) but was less specific for the temporal separation (indicator value < 0.6) (Table S4). Of the other eukaryotic OTUs, the distributions of OTU3, OTU6, and OTU7 were specifically observed during summer (June and August) in 2016 (indicator value > 0.6, *P* < 0.01), and OTU10 was specifically distributed in February 2017 (indicator value > 0.6, *P* < 0.01) (Table S4). These eukaryotic OTUs were taxonomically assigned to Dinoflagellata (genera *Karenia* [OTU1] and *Noctiluca* [OTU2]), Diatomea (genera *Chaetoceros* [OTU3, OTU5, and OTU7], *Thalassiosira* [OTU4], and *Cyclotella* [OTU6]), and Ciliophora (genera *Didinium* [OTU8] and *Pseudotontonia* [OTU10]). Interactions among these prokaryotic and eukaryotic OTUs with environmental factors were evaluated using the spearman correlation, and their co-occurrence patterns were visualized in the network association (Fig. 6). The network co-occurrence analysis revealed that most of prokaryotic OTUs except for two network nodes (OTU3 and OTU5) were widely connected with other OTUs. In the network association, the isolated nodes with no edge indicate none of interaction at significant level (correlation coefficient < 0.5, *P* > 0.05).

## Discussion

In GB waters, diversity and community composition of prokaryotes and microeukaryotes was surveyed at the spatiotemporal scale using the 16S and 18S rRNA gene metabarcoding approach. The biogeographic patterns of microbial communities represent the environmental heterogeneity at the spatiotemporal scale in the estuarine–coastal zone. The indices of alpha diversity revealed that the estuarine and coastal water types have similar levels of species richness in both prokaryotic and eukaryotic communities and that the microbial species richness is in response to temperature change, indicating the seasonality differences. Moreover, the patterns of beta diversity observed from NMDS and AMOVA supported that the prokaryotic and eukaryotic communities in GB waters are distinguishable at the spatiotemporal scale. In particular, the local community compositions in response to the spatiotemporal separations were represented by the population dynamics of major prokaryotic and eukaryotic taxa.

The taxonomic assignments of eukaryotic OTU1 (*Karenia*) and OTU2 (*Noctiluca*) are known to be harmful blooming players in Dinoflagellata^33,34^. *Noctiluca*, a bioluminescent organism (sea sparkle), is a large (0.2–2 mm) non-photosynthetic dinoflagellate that feeds on bacteria and small size dinoflagellates, diatoms, and zooplankton eggs^35^, whereas *Karenia* (eukaryotic OTU1) is a small (20–90 μm) dinoflagellate. The occurrence of OTUs related to *Karenia* (May and June) and *Noctiluca* (November) in GB waters followed their general blooming trend in oceans. In eukaryotic OTUs related to Diatomea (2–200-μm size of blooming organisms), *Chaetoceros* (OTU3, OTU5, and OTU7) is known to be a common phytoplankton during summer in Korean coastal water^36^, as shown in this study, and the occurrence of other Diatomea populations such as *Thalassiosira* (OTU4), *Rhizosolenia* (OTU10), and *Cyclotella* (OTU6) was previously documented in Korea^37–39^. In addition, *Didinium* (OTU8) showed fresh or brackish water-specific distribution, and *Pseudotontonia* (OTU9) was ubiquitous in GB waters.

The network co-occurrence analysis for prokaryotic and eukaryotic OTUs implies their biological interactions including predation, cross-feeding, mutualism, and competition. For example, the strong positive interaction between heterotrophic bacterium (prokaryotic OTU1) and protist (eukaryotic OTU8) suggests a trophic role of protozoans, responsible for energy transfer from primary producers (bacteria and phytoplankton) to fish in the marine food web system. Similarly, a photosynthetic dinoflagellate (eukaryotic OTU1) showed interactions with heterotrophic Roseobacter clade (OTU4) and SAR86 clade (OTU9) bacteria, which possess metabolic pathways for degrading phytoplankton-derived organic matter^40,41^. Furthermore, the strong negative relation between heterotrophic bacterium (prokaryotic OTU2) and cyanobacterium (prokaryotic OTU7) implies bacterial degradation of cyanobacterial necromass^42^. In contrast to prokaryotic nodes, eukaryotic OTUs were more widely connected with environmental factors rather than OTU nodes. Only two eukaryotic OTUs (OTU1 and OTU8) were strongly linked to other OTUs, and most of eukaryotic OTUs were weakly connected or isolated in the network. In addition, we also found the interactions between ChlA and phytoplanktons (Cyanobacteria, Diatom, Dinoflagellata) (Table S5), but they were removed from the network association due to their weak correlation coefficient (< 0.5).

Interest of ecological processes influencing microbial community assembly has increased in various environments^16,23–29,32^. The previous GB survey^16^ provides an understanding of the phylogenetic structuring of bacterial communities in the estuarine–coastal zone. Although the previous study revealed that the assembly process of bacterial communities, which exclude archaeal populations from prokaryotic communities, is more influenced by phylogenetic clustering within euphotic layers (< 13 m depth), the comparison of prokaryotic communities (including both bacterial and archaeal populations) with the assembly of eukaryotic communities may need to be considered within the surface (or atmospheric) mixed layer (< 0.3 m depth) having nearly constant environmental conditions. Thus, we newly analyzed the assembly process of prokaryotic and eukaryotic communities. The predominant patterns of phylogenetic structuring in prokaryotic (over-dispersed type: biotic interaction) and eukaryotic (clustered type: abiotic interaction) communities were remarkably observed in coastal waters, suggesting that prokaryotes and eukaryotes follow different strategies in community assembly at least in coastal waters. For example, strong biological interactions were expected to be the main driving force in the prokaryotic community assembly, whereas eukaryotes were more affected by abiotic factors in coastal waters. On the other hand, there was no primary signal from NTR–NTI patterns for both prokaryotic and eukaryotic communities in estuarine waters. Similarly, the balance between deterministic and stochastic processes revealed that prokaryotes and eukaryotes have different community assembly processes in coastal waters although they are primarily influenced by the stochastic process in estuarine waters. Our results suggest that the assembly process of prokaryotic communities was primarily affected by the stochastic process (facilitation and competition among their populations), but the community assembly process for eukaryotic populations was relatively more affected by environmental stress than by the deterministic process (biological interaction) in coastal waters. Indeed, these phylogenetic diversity patterns were supported by distinguishable interactions within prokaryotic or eukaryotic OTUs in the microbial association network. Based on our findings, it can be concluded that prokaryotic and eukaryotic communities in GB waters possess a distinguishable mechanism in their community assembly, although they simultaneously represent the spatiotemporal separation in the estuarine–coastal zone.

Transition of microbial communities in the estuarine–coastal zone may provide information of key microbial groups indicating the mixing from the estuarine to coastal waters. The previous GB survey by Han et al.^16^ found dominant bacterial groups showing the gradual distribution from the estuarine to coastal waters with seasonality. Of their spatiotemporal distribution, the SAR11 clade (*Candidatus Pelagibacter ubique*), a ubiquitous heterotrophic bacterium in oceans^43^, revealed the gradually increasing variation toward coastal waters, representing the water mass mixing in the estuarine–coastal zone. The SAR11 clade could tolerate a wide range of environmental variability including salinity during the water mass mixing due to its oligotrophic lifestyle and the ecological interface with phytoplankton^44^. In the present study, we found that the most predominant prokaryotic OTU (OTU1) in coastal waters was taxonomically identified as a member of the SAR11 clade. The prevalence of prokaryotic OTU1 associated with SAR11 in this study implies the bacterial degradation of phytoplankton-derived organic matter in GB waters as previously reported^16^. Indeed, the most dominant eukaryotic OTUs were found to be affiliated with phytoplankton (Diatomea or Dinoflagellata). It was known that phytoplankton-derived organic matter can attract heterotrophic bacteria^45^, and specifically associated bacteria are beneficial for the survival of phytoplankton hosts^46^. Taken together with the specificity of phytoplankton-bacteria interactions, the co-occurrence of heterotrophic bacterial and phytoplankton OTUs in the microbial association network may support trophic interactions in microbial communities in GB waters.

In this study, we demonstrated the use of metabarcoding approach in surveying microbial biogeography associated with environmental heterogeneity in the estuarine–coastal zone. We surveyed prevalent prokaryotic and eukaryotic taxa in GB waters from 2016 to 2018. Here, phytoplankton sequences were found to be dominant in GB waters and their spatiotemporal distribution was observed. Considering the similar distribution between heterotrophic Proteobacteria and phytoplankton at the spatiotemporal scale in the present study, our findings at 2- to 4-month intervals from 2016 to 2018 may imply the ecological interaction in response to the environmental heterogeneity mediated by seasonal climate change rather than annual change in the estuarine–coastal zone. Information on the microbial biogeography and its community assembly process can provide fundamental knowledge regarding the given ecological system. The fate of heterotrophic prokaryotes in coastal waters is generally associated with factors regulating nutrient flux, including phytoplankton-derived organic matter. Bacterial contribution to oceanic nutrient dynamics was previously studied in GB waters^16^. However, the ecological significance of eukaryotic communities compared with that of prokaryotic communities in this region has never been reported. Here, we proposed ecological interactions microeukaryotes (phyto- and zooplankton) and heterotrophic bacteria such as SAR11 based on their network association. We believe that our study newly facilitates the assessment of ecological significance in microbial trophic interactions and of underlying biotic or abiotic factors for the assembly process of microbial communities in the estuarine–coastal zone.

## Methods

### Description of study area in GB and datasets

In the current climate condition, GB waters generally show seasonal separations with gradients of temperature and salinity due to the water mass mixing between estuarine and coastal waters. Long-Term Marine Ecological Research (LTMER) designed nine sampling stations with a spatially similar distance in GB (Fig. 1a) to monitor environmental heterogeneity in the estuarine-coastal zone and spatiotemporal biogeography of microbial communities^16^. Experimental designs and sample collection to construct dataset for the GB monitoring were previously described^16^. Briefly, temperature, salinity, PO_4_, NH_4_, NO_2_, NO_3_, SiO_2_, and ChlA measured to estimate the environmental heterogeneity in GB. For the microbial biogeographic survey, one liter of seawater (< 0.3 m depth) was collected from each GB station at 2- to 4-month intervals from 2016 to 2018 and immediately filtered using 0.2 μm hydrophilic PVDF membranes (Merck, Darmstadt, Germany). The filtered membranes were kept at − 80 °C before extraction of environmental DNAs (eDNAs).

The present study complied nine datasets (metadata and sequence) of GB surface seawaters obtained at < 0.3 m depth in: (1) August, (2) June, and (3) November, 2016; (4) February and (5) June, 2017; (6) March, (7) May, (8) November, and (9) September, 2018 (Table 1). Most of datasets (metadata and their prokaryotic and eukaryotic sequences) constructed from this study. Five datasets from 2016 and 2017 were selectively consisted of metadata from the previous study^16^ and their prokaryotic 16S rRNA metabarcoding sequences from European Nucleotide Archive (https://www.ebi.ac.uk/ena) under the accession number ERP110504.

**Table 1:** Description of datasets in this study.

Data description	Number of samples	Sampled date (month, year)	Target gene	Data source
Metabarcoding sequences using prokaryotic 16S rRNA gene	9	June, 2016	16S rRNA gene (V3–V4 region)	Han et al*.*^16^
	9	August, 2016	16S rRNA gene (V3–V4 region)	Han et al*.*^16^
	9	November, 2016	16S rRNA gene (V3–V4 region)	Han et al*.*^16^
	9	February, 2017	16S rRNA gene (V3–V4 region)	Han et al*.*^16^
	9	June, 2017	16S rRNA gene (V3–V4 region)	Han et al.^16^
	9	March, 2018	16S rRNA gene (V3–V4 region)	This study
	8	May, 2018	16S rRNA gene (V3–V4 region)	This study
	8	September, 2018	16S rRNA gene (V3–V4 region)	This study
	9	November, 2018	16S rRNA gene (V3–V4 region)	This study
Metabarcoding sequences using eukaryotic 18S rRNA gene	9	June, 2016	18S rRNA gene (V8–V9 region)	This study
	9	August, 2016	18S rRNA gene (V8–V9 region)	This study
	9	November, 2016	18S rRNA gene (V8–V9 region)	This study
	9	February, 2017	18S rRNA gene (V8–V9 region)	This study
	9	June, 2017	18S rRNA gene (V8–V9 region)	This study
	9	March, 2018	18S rRNA gene (V8–V9 region)	This study
	9	May, 2018	18S rRNA gene (V8–V9 region)	This study
	8	November, 2018	18S rRNA gene (V8–V9 region)	This study

### Extraction of eDNA and sequence data processing

eDNAs were extracted from the frozen membranes using PowerWater DNA Isolation Kit (MOBIO Laboratories, Carlsbad, CA, USA) and further proceeded for metabarcoding-based sequencing with two-step PCRs (amplicon and index PCR) according to the previously described protocol^16^. First, eDNAs were PCR-amplified with primers specific to prokaryotic 16S^47^ and eukaryotic 18S^10^ rRNA genes (the amplicon PCR). The amplicon PCR was performed using the following program: (1) 95 °C for 3 min, (2) 25 cycles of 95 °C for 30 s, 55 °C for 30 s, and 72 °C for 30 s, and (3) 72 °C for 5 min. The amplified rRNA gene fragments were used as template DNAs for the further index PCR. The index PCR was performed using the following program: (1) 95 °C for 3 min, (2) 8 cycles of 95 °C for 30 s, 55 °C for 30 s, and 72 °C for 30 s, and (3) 72 °C for 5 min. Amplicons of the index PCRs were purified, and their concentrations were measured by Qubit 2.0 Fluorometer (Invitrogen, Carlsbad, CA, USA). The purified PCR amplicons were mixed in equimolar amounts to construct a MiSeq library, and the final PCR mixture was subjected to the metabarcoding sequencing using the Illumina MiSeq platform (Macrogen, Seoul, South Korea). Details of the two-step PCRs and the construction of the MiSeq library are described in the Illumina’s instruction manual^48^, and the used primer sequences are listed in the Supplementary Information 2 (see extra description). The obtained metabarcoding sequences were submitted to the National Center for Biotechnology Information (NCBI) Sequence Read Archive (https://www.ncbi.nlm.nih.gov/sra) under the accession number PRJNA669608 (prokaryotic 16S rRNA gene) and PRJNA669603 (eukaryotic 18S rRNA gene).

The sequencing data were analyzed using the Mothur software (v.1.40.5)^49^ based on the MiSeq SOP^50^. Briefly, quality filtering of sequences was performed with (1) correction of amplification and sequencing errors (removing chimeric sequences, (2) singleton removal, and (3) random subsampling of sequences. In particular to the random subsampling, the sequence count number for each individual sample was normalized with 13,000 sequences of prokaryotic 16S rRNA gene and 11,000 sequences of eukaryotic 18S rRNA gene, respectively. These filtered sequences were clustered into the OTUs at 97% similarity level to investigate microbial diversity (alpha and beta), community composition, and phylogenetic turnover.

### OTU-based analyses and statistics

For microbial alpha diversity (species richness), abundance-unweighted species richness indices (Chao1 and ACE) were calculated with OTUs using the ‘summary.single’ command, while beta diversity was determined by NMDS and AMOVA calculated using the ‘nmds’ and ’amova’ commands, respectively. In addition, microbial community composition was profiled using the ‘classify.otu’ command against the Silva.seed_v132 database in Mothur. Phylogenetic diversity was analyzed using Phylocom software^51^ based on the protocol described previously^32^. Briefly, phylogenetic structure of microbial communities was estimated with NRI and NTI, calculated using the ‘comstruct’ command, whereas βNTI was calculated using ‘comdistnt’ command to determine the influence of deterministic and stochastic processes.

We performed statistical analyses using R software (v.3.5.3) (https://www.R-project.org). From the R stats package^52^, for example, PCA was carried out using the ‘prcomp’ function, and correlation analysis was applied with spearman method using the ‘cor’ function. From the vegan package^53^, PERMANOVA and NMDS were calculated by Bray–Curtis distance with the ‘adonis’ and ‘metaMDS’ functions, respectively. In addition to PERMANOVA, the pairwise multiple comparison (post-hoc) was performed with false discovery rate method in the ‘pairwise.adonis’ function^54^. Indicator species analysis was carried out using the ‘indval’ function in the labdsv package^55^. Association of OTU variations at the spatiotemporal scale was estimated with a heatmap visualization using the ‘HeatmapAnnotation’ function in the ComplexHeatmap package^56^. Microbial network interactions with environmental factors were analyzed using the RCy3 package^57^ with Cytoscape software (v.3.8.2) (https://cytoscape.org). Briefly, the spearman correlation matrix was calculated with the relative abundance of prokaryotic and eukaryotic OTUs and measured values of environmental factors. Co-occurrence patterns were determined with significant coefficient values over 0.5 (*P* < 0.05) in the spearman correlation. The significant correlations between OTUs or between OTU and environmental factor were visualized in network interactions.

## Supplementary Information

Supplementary Information 1.

Supplementary Information 2.
