# Supplementary material for: Phylogenetic diversity and spatiotemporal dynamics of bacterial and microeukaryotic plankton communities in Gwangyang Bay of the Korean Peninsula
Source: Sci Rep. 2022 Feb 22;12:2980. doi: 10.1038/s41598-022-06624-7 (PMC8863807; doi:10.1038/s41598-022-06624-7)
Supplement: Supplementary file 2 — Supplementary Information 2. [file 41598_2022_6624_MOESM2_ESM.docx]

**Title:** Phylogenetic Diversity and Spatiotemporal Dynamics of Bacterial and Microeukaryotic Plankton Communities in Gwangyang Bay of the Korean Peninsula

Dukki Han^1^*, Hanseob Shin^2^, Ji-Hoon Lee^3^, Chang-Keun Kang^2^, Dong-Gyun Kim^4^, and Hor-Gil Hur^2^

^1^Department of Marine Molecular Bioscience, Gangneung-Wonju National University, 7, Jukheon-gil, Gangneung-si, Gangwon-do 25457, Republic of Korea; ^2^School of Earth Sciences and Environmental Engineering, Gwangju Institute of Science and Technology, 123 Cheomdangwagi-ro, Buk-gu, Gwangju 61005, Republic of Korea; ^3^Department of Bioenvironmental Chemistry, Jeonbuk National University, Jeonju, Republic of Korea; ^4^Biotechnology Research Division, National Institute of Fisheries Science, Busan, 46083, Republic of Korea.



**Running title:** Microbial phylogenetic diversity in coastal water

**^*^For correspondence:** D. Han (dukkihan@gwnu.ac.kr)

**Extra description**: Ecological terminology

| **Ecological terminology** | **Description** | **Reference** |
| --- | --- | --- |
| Deterministic process | Assembly process of community composition is influenced by abiotic factor (environmental selection) and species interaction (antagonistic and synergistic). Deterministic process can be categorized into homogeneous (low compositional turnover) or variable selections (high compositional turnover). | (Stegen et al. 2015) |
| Stochastic process | Assembly process of community composition is under unpredictable disturbance in species movement (ecological drift or dispersal) or population size. Stochastic process can be separated into dispersal limitation or homogenous dispersal. | (Stegen et al. 2015) |
| The net relatedness index (NRI) | “A standardized measure of the mean pairwise phylogenetic distance of taxa in a sample, relative to a phylogeny of an appropriate species pool, and quantifies overall clustering of taxa on a tree” | (Webb et al. 2002) |
| The nearest taxon index (NTI) | “A standardized measure of the phylogenetic distance to the nearest taxon for each taxon in the sample and quantifies the extent of terminal clustering, independent of deep level clustering” | (Webb et al. 2002) |
| Beta Nearest Taxon Index (βNTI) | A measure of the phylogenetic beta diversity between samples (the between-assemblage analogs of NTI) | (Webb et al. 2002) |
| Phylogenetic turnover model | Homogeneous selection in deterministic process: significantly less than expected phylogenetic turnover (βNTI < -2); variable selection in deterministic process: significantly more than expected phylogenetic turnover (βNTI > +2); stochastic process: the lack of deviation (-2 < βNTI < +2) | (Dini-Andreote et al. 2015; Stegen et al. 2012) |

**Extra description:** Information of used primers in this study.

| Primer | Target region | Sequence (5' ***Illumina’s adapter region***─target primer sequence 3´) | Reference |
| --- | --- | --- | --- |
| Bakt_341F | 16S rRNA gene V3 | 5´ ***TCGTCGGCAGCGTCAGATGTGTATAAGAGACAG***─CCTACGGGNGGCWGCAG 3´ | (Herlemann et al. 2011) |
| Bakt_805R | 16S rRNA gene V4 | 5´***GTCTCGTGGGCTCGGAGATGTGTATAAGAGACAG***─GACTACHVGGGTATCTAATCC 3´ |  |
| V8f | 18S rRNA gene V8 | 5´ ***TCGTCGGCAGCGTCAGATGTGTATAAGAGACAG***─ATAACAGGTCTGTGATGCCCT 3´ | (Agogué et al. 2011) |
| 1510r | 18S rRNA gene V9 | 5´***GTCTCGTGGGCTCGGAGATGTGTATAAGAGACAG***─CCTTCYGCAGGTTCACCTAC 3´ |  |


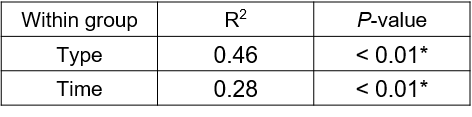
Table S1. Statistical separation of GB waters with environmental parameters supported by PERMANOVA. Significant differences within group or between pairs were marked with an asterisk (*P* < 0.01).


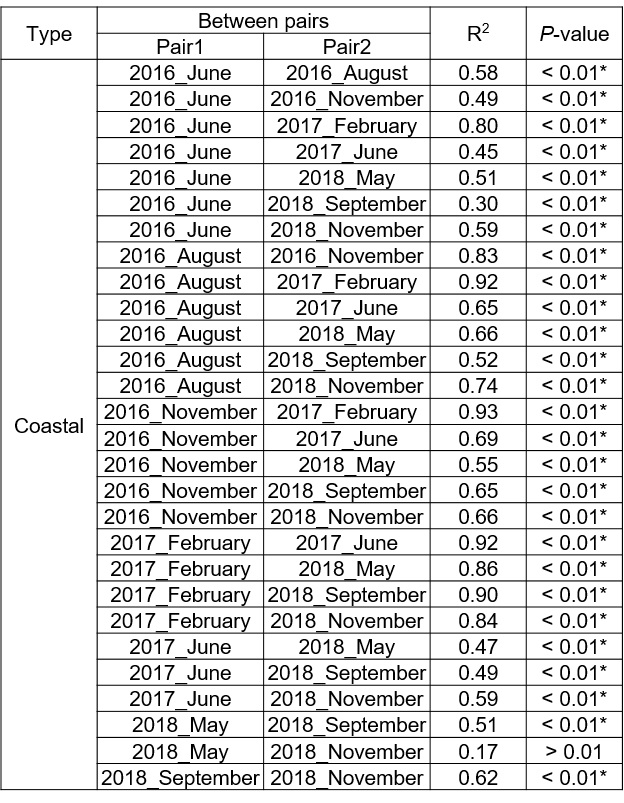

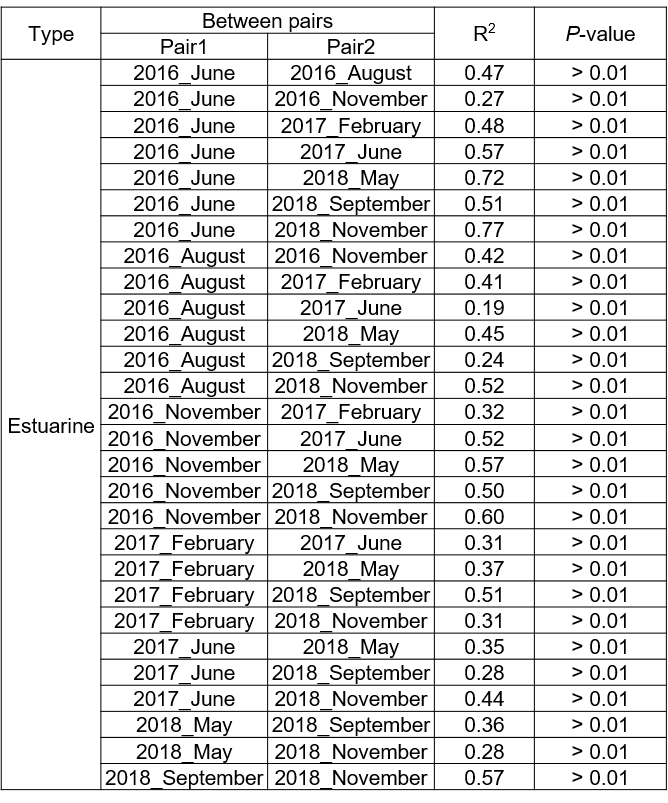


Table S2. Statistical separation among prokaryotic and eukaryotic communities supported by AMOVA. Significant differences within group or between pairs were marked with an asterisk (*P* < 0.01).


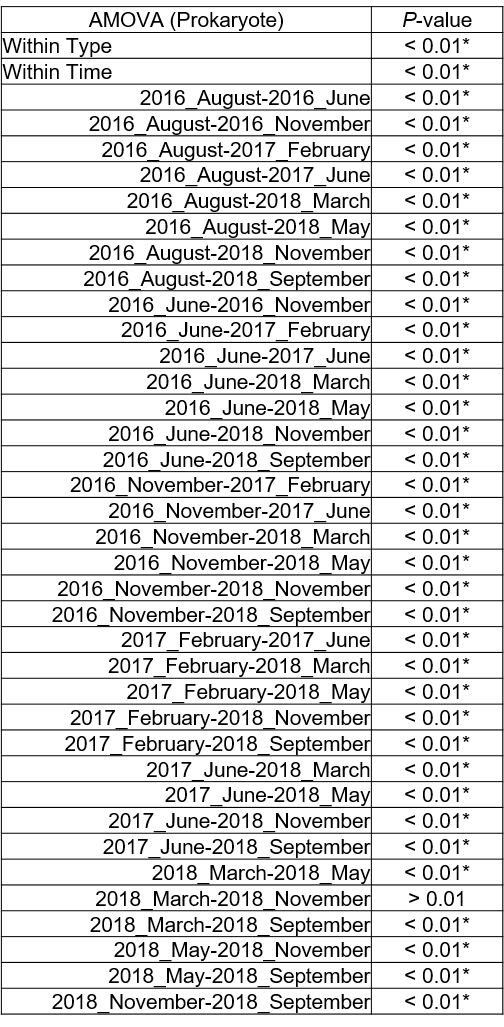

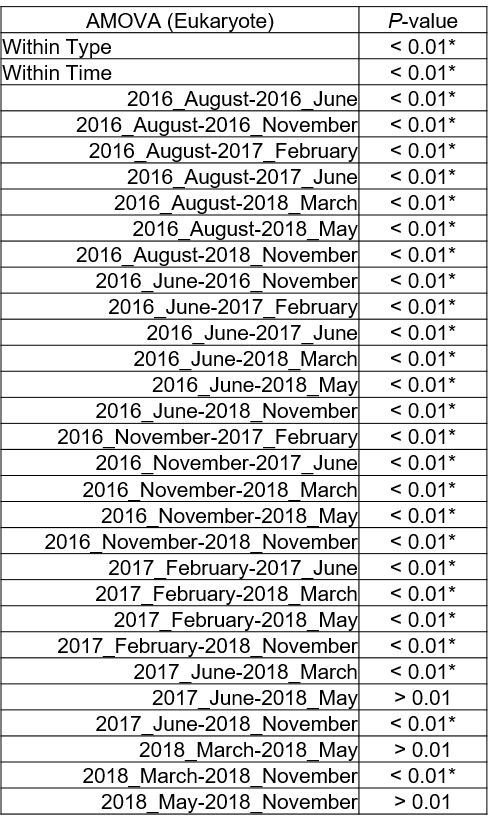


| Prokaryotes (Phylum) |  | Temp.: | |  | Salinity: | |
| --- | --- | --- | --- | --- | --- | --- |
|  |  | Cor. | *P*-value |  | Cor. | *P*-value |
| Proteobacteria |  | 0.13 | > 0.01 |  | **0.31** | **< 0.01** |
| Bacteroidota |  | 0.20 | > 0.01 |  | **-0.43** | **< 0.01** |
| Actinobacteriota |  | **0.29** | **< 0.01** |  | **-0.63** | **< 0.01** |
| Verrucomicrobiota |  | -0.16 | > 0.01 |  | 0.19 | > 0.01 |
| Cyanobacteria |  | 0.18 | > 0.01 |  | -0.11 | > 0.01 |
| Planctomycetota |  | -0.07 | > 0.01 |  | 0.09 | > 0.01 |
| Firmicutes |  | **0.42** | **< 0.01** |  | -0.16 | > 0.01 |

Table S3. Correlation (Spearman) between the relative abundance of phylum taxa and gradient of temperature and

Salinity. Significant correlations were marked in bold (*P* < 0.01)

| Eukaryotes (Phylum) |  | Temp.: | |  | Salinity: | |
| --- | --- | --- | --- | --- | --- | --- |
|  |  | Cor. | *P*-value |  | Cor. | *P*-value |
| Diatomea |  | 0.01 | > 0.01 |  | 0.05 | > 0.01 |
| Dinoflagellata |  | -0.17 | > 0.01 |  | **0.53** | **< 0.01** |
| Ciliophora |  | -0.11 | > 0.01 |  | -0.1 | > 0.01 |
| Ascomycota |  | 0.25 | > 0.01 |  | -0.25 | > 0.01 |
| Cryptophyceae |  | 0.03 | > 0.01 |  | -0.17 | > 0.01 |
| Chlorophyta |  | **0.33** | **< 0.01** |  | **-0.49** | **< 0.01** |
| Ochrophyta |  | -0.1 | > 0.01 |  | -0.26 | > 0.01 |
| Cnidaria |  | 0.11 | > 0.01 |  | **0.35** | **< 0.01** |

Table S4. Indicator species analysis using prokaryotic and eukaryotic OTUs. Significance of indicator value was represented with an asterisk (*P* < 0.01).


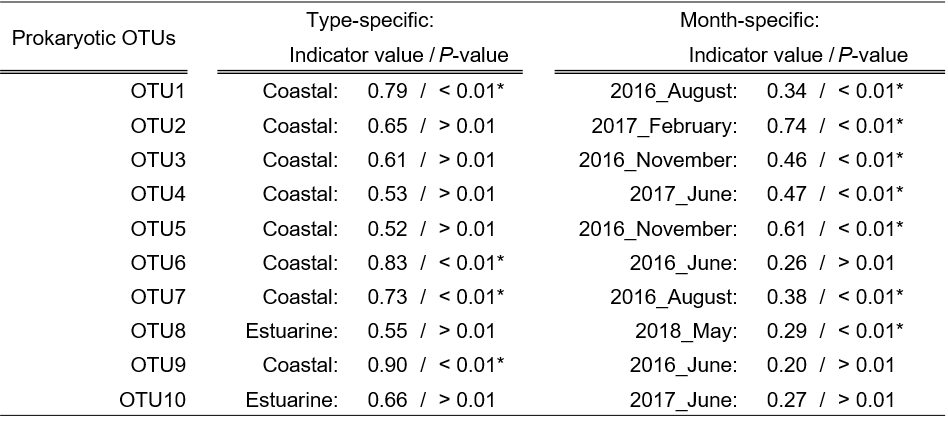

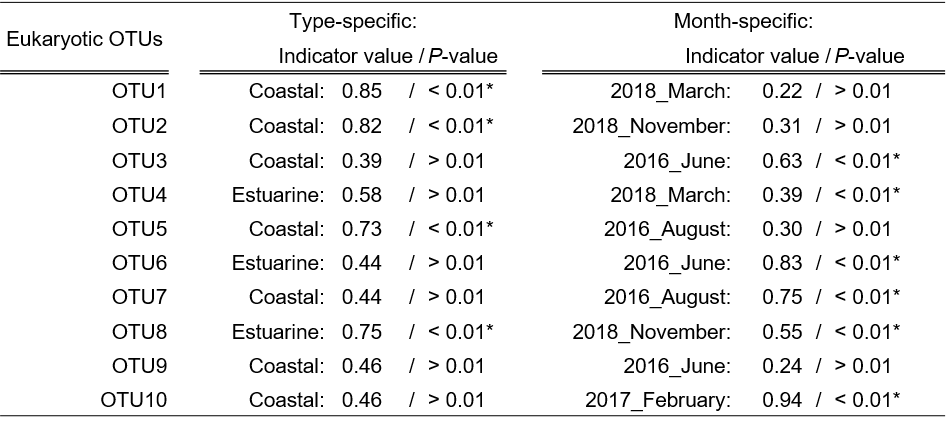


Table S5. Connection between ChlA and phytoplanktons (Cyanobacteria, Diatom, Dinoflagellata) in the network co-occurrence analysis.

| OTUs | Taxa | Env. | Cor. (*P* < 0.05) |
| --- | --- | --- | --- |
| Eukaryotic.OTU4 | Thalassiosira (Diatom) | ChlA | 0.44 |
| Eukaryotic.OTU10 | Rhizosolenia (Diatom) | ChlA | 0.43 |
| Eukaryotic.OTU6 | Cyclotella (Diatom) | ChlA | 0.34 |
| Prokaryotic.OTU7 | Synechococcus (Cyanobacteria) | ChlA | -0.29 |
| Eukaryotic.OTU2 | Noctiluca (Dinoflagellata) | ChlA | -0.31 |
| Eukaryotic.OTU7 | Chaetoceros (Diatom) | ChlA | -0.45 |

Fig. S1. Alpha diversity of prokaryotic and eukaryotic communities represented by unweighted species richness estimators (Chao1 and Ace) in GB waters.


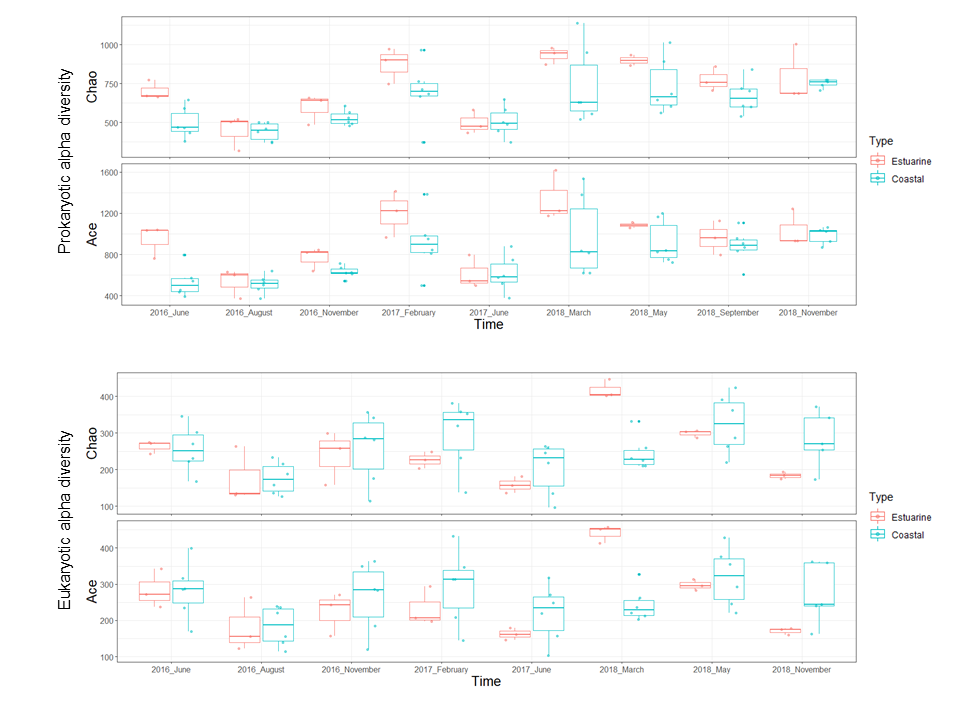


Fig. S2. Beta diversity of prokaryotic and eukaryotic communities visualized by non-metric multi-dimensional scaling.


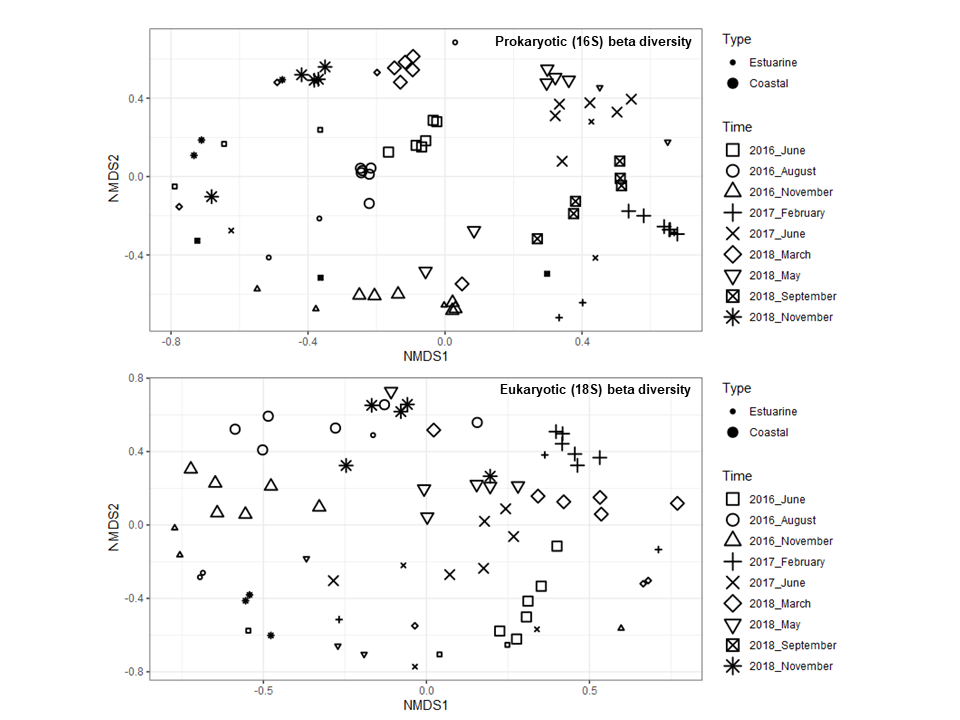


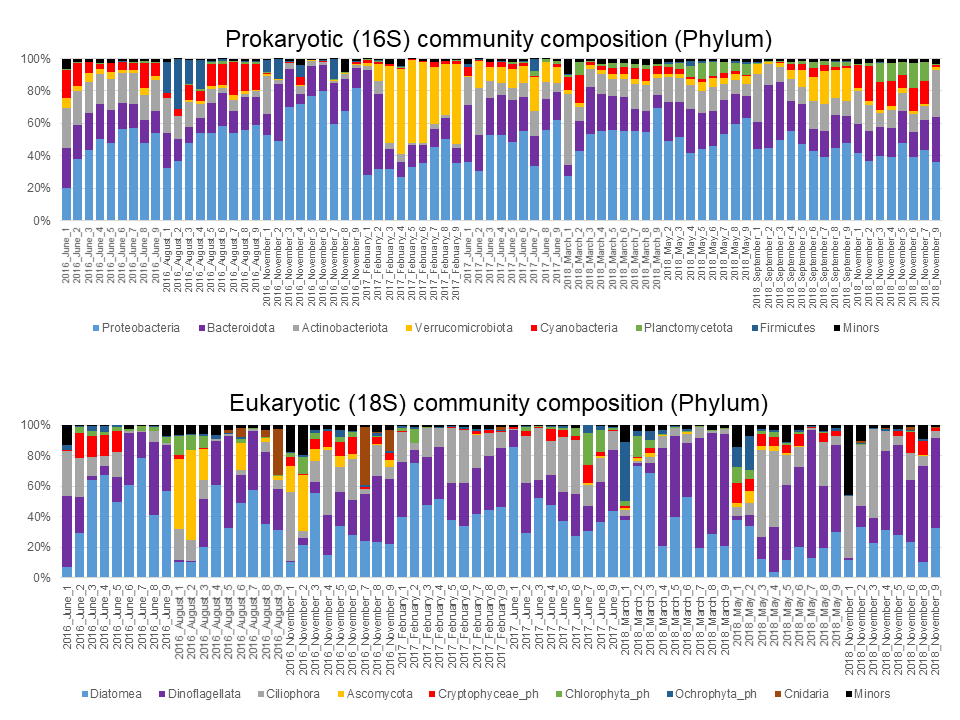
Fig. S3. Prokaryotic and eukaryotic community composition at phylum level. Relative abundance of whole communities was listed in Supplementary data (excel file)

Relative abundance

Relative abundance

**References**

Agogué, H., D. Lamy, P.R. Neal, M.L. Sogin, and G.J. Herndl. 2011. Water mass‐specificity of bacterial communities in the North Atlantic revealed by massively parallel sequencing. *Molecular ecology* 20: 258-274.

Dini-Andreote, F., J.C. Stegen, J.D. van Elsas, and J.F. Salles. 2015. Disentangling mechanisms that mediate the balance between stochastic and deterministic processes in microbial succession. *Proceedings of the National Academy of Sciences* 112: E1326-E1332.

Herlemann, D.P., M. Labrenz, K. Jürgens, S. Bertilsson, J.J. Waniek, and A.F. Andersson. 2011. Transitions in bacterial communities along the 2000 km salinity gradient of the Baltic Sea. *The ISME journal* 5: 1571-1579.

Stegen, J.C., X. Lin, J.K. Fredrickson, and A.E. Konopka. 2015. Estimating and mapping ecological processes influencing microbial community assembly. *Frontiers in microbiology* 6: 370.

Stegen, J.C., X. Lin, A.E. Konopka, and J.K. Fredrickson. 2012. Stochastic and deterministic assembly processes in subsurface microbial communities. *The ISME journal* 6: 1653-1664.

Webb, C.O., D.D. Ackerly, M.A. McPeek, and M.J. Donoghue. 2002. Phylogenies and community ecology. *Annual review of ecology and systematics* 33: 475-505.
